# Cost‐Effectiveness Analysis of an Intensive Lifestyle Intervention Versus Usual Care for Latino Youth With Prediabetes

**DOI:** 10.1111/ijpo.70046

**Published:** 2025-07-31

**Authors:** Armando Peña, Micah L. Olson, Mary Beth Putz, Abu Bakkar Siddique, Elvia Lish, Monica Diaz, Stephanie L. Ayers, J. Mac McCullough, Allison Williams, Donald L. Patrick, Gabriel Q. Shaibi

**Affiliations:** ^1^ School of Public Health‐Bloomington Indiana University‐Bloomington Bloomington Indiana USA; ^2^ Division of Pediatric Endocrinology and Diabetes Phoenix Children's Hospital Phoenix Arizona USA; ^3^ Center for Health Promotion and Disease Prevention Arizona State University Tempe Arizona USA; ^4^ Phoenix Children's Pediatric Residency Program Alliance Phoenix Arizona USA; ^5^ School of Public Administration Florida Atlantic University Boca Raton Florida USA; ^6^ Ivy Center for Family Wellness The Society of St. Vincent de Paul Phoenix Arizona USA; ^7^ Southwest Interdisciplinary Research Center Arizona State University Phoenix Arizona USA; ^8^ School of Public and Population Health Boise State University Boise Idaho USA; ^9^ School of Public Health University of Washington Seattle Washington USA

**Keywords:** cost‐effectiveness, Latino, lifestyle intervention, prediabetes, youth

## Abstract

**Objective:**

Lifestyle intervention is a cost‐effective approach for preventing type 2 diabetes among adults with prediabetes. The purpose of this study was to examine the cost and incremental cost‐effectiveness ratio (ICER) of an intensive lifestyle intervention compared with usual care among youth with prediabetes.

**Methods:**

Latino youth ages 12–16 years with obesity and prediabetes were randomised to a 6‐month lifestyle intervention (INT, *N* = 79) or usual care (UC, *N* = 38). Between‐group difference in change in 2‐h post‐challenge glucose was non‐significant at 6 months (7.2 mg/dL; 95% CI, −5.3, 19.7) and 12 months (0.3 mg/dL; 95% CI, −14.1, 14.5). Cost and ICERs were calculated from health care, family and total societal (health care plus family) perspectives in 2019 US Dollars. The study was conducted from May 2016 to March 2020.

**Results:**

The estimated societal cost per participant was $1935.21 (95% CI, $1583.12, $2315.05) for the INT and $234.20 ($190.71, $278.61) for the UC during the first 6 months. During the entire 12 months of the trial, the societal cost per participant increased to $468.40 ($379.19, $568.37) for the UC, but there were no additional costs for the INT. The ICER for 2‐h glucose for the INT compared with the UC was $222.78 and $4816.57 per mg/dL at 6 and 12 months.

**Conclusions:**

Among Latino youth with prediabetes, an intensive lifestyle intervention resulted in a nonsignificant reduction in 2‐h glucose at a higher cost compared with usual care.

## 1 Introduction

The number of adolescents in the United States with prediabetes or type 2 diabetes (T2D) is increasing [1, 2]. The diagnosis of T2D at a young age has a significant impact on future health, as paediatric T2D is associated with rapid deterioration of pancreatic beta‐cell function [3, 4], accelerated onset of diabetes‐related complications [5] and premature mortality [6]. In addition to the health consequences, paediatric T2D has important economic implications, with a younger age at diagnosis leading to a longer lifetime economic burden [7, 8].

The Diabetes Prevention Program (DPP) was a landmark clinical trial, first published in 2002, that demonstrated that lifestyle intervention is a cost‐effective approach for reducing the incidence of diabetes in adults with prediabetes [9, 10, 11, 12, 13]. Based largely on the strength of the DPP results, lifestyle intervention is recommended as the first‐line approach for preventing T2D among adults at high risk [14].

Numerous studies have evaluated the clinical efficacy of intensive lifestyle intervention on weight outcomes for paediatric obesity, and a number of these trials have associated cost‐effectiveness analyses [15, 16, 17, 18]. To our knowledge, only one previous study has tested whether intensive lifestyle intervention can lead to diabetes risk reduction in children with prediabetes [19]. This was a study of the Bright Bodies Healthy Lifestyle Program on youth with impaired glucose tolerance, which reported a robust reduction in 2‐h glucose (−17.1 mg/dL, *p* = 0.005) in response to an oral glucose tolerance test (OGTT) compared with standard clinical care. The Bright Bodies program has published a cost‐effectiveness analysis associated with changes in weight outcomes, but not related to their diabetes risk reduction study [16]. Therefore, we are not aware of any previously published economic analysis of paediatric diabetes prevention interventions.

To build on this work, we conducted a two‐arm randomised clinical trial among Latino youth with prediabetes that demonstrated reductions in diabetes risk factors across both study arms [20]. Despite the need for diabetes prevention programmes among youth with prediabetes, whether such programmes are cost‐effective in the paediatric population is not known. Measuring the cost‐effectiveness of lifestyle interventions demonstrates the economic value of such interventions which, in turn, informs decisions regarding their development, implementation and reimbursement. Therefore, the purpose of this study was to examine the cost and incremental cost effectiveness of an intensive lifestyle intervention compared with usual care for reducing diabetes risk among youth with prediabetes.

## 2 Methods

### 2.1 Participants and Intervention

The Preventing Diabetes in Latino Youth study was a 12‐month randomised clinical trial that tested the efficacy of an intensive lifestyle programme compared with a usual care comparison group for reducing diabetes risk factors among Latino youth with prediabetes. The study protocol and primary outcomes have been published elsewhere [20, 21] and are briefly summarised below.

Participants were selected for the following inclusion criteria: (1) self‐reported Latino descent, (2) ages 12–16 years, (3) body mass index (BMI) ≥ 95th for age and sex, and (4) prediabetes as defined by HbA1c 5.7%–6.4%, fasting glucose 100–125 mg/dL or 2‐h glucose 120–199 mg/dL following a 75‐g OGTT, but not meeting any of the criteria for diabetes.

Study participants were randomised in a 2:1 fashion to a lifestyle intervention (INT) or a usual care (UC) comparison group. The INT was a 6‐month lifestyle program that included 1 day/week of nutrition and health education with behaviour change skills training and 3 days/week of physical activity. The education and activity sessions were delivered at two local YMCA sites. Health education sessions were delivered by bilingual, bicultural community health educators from a local community clinic to groups of 8–10 families and promoted the adoption of a healthy balanced diet, including reducing intake of saturated fat, added sugars and sugar‐sweetened beverages, increasing intake of fibre, fruit and vegetables, as well as managing portion sizes. Participants set weekly individual health behaviour goals using the Specific, Measurable, Attainable, Relevant and Timely (SMART) goal framework. Physical activity was delivered by YMCA instructors twice per week for 60 min/session and included circuit training, sports activities (e.g., basketball, soccer), agility and cardiovascular exercises. A third day of independent physical activity was promoted and tracked by instructors on a weekly basis to complete a minimum of 180 min of moderate‐to‐vigorous physical activity per week.

The UC arm consisted of two 1‐h visits with a paediatric endocrinologist and a bilingual, bicultural registered dietitian to discuss laboratory results and develop SMART goals for making healthy lifestyle changes. The first visit occurred shortly after randomization and the second visit occurred at 6 months. The visits were designed to mirror the care provided to youth with obesity who are managed at the Phoenix Children's Endocrine Division programme for weight‐related disorders. The visits were held at the Arizona State University (ASU) campus.

This study was approved by the ASU Institutional Review Board and in accordance with the Declaration of Helsinki. Youth provided written assent and parents provided written consent before study participation. Enrolment began in May 2016 and ended in June 2019.

### 2.2 Health Outcomes

Data were collected at baseline (T1), 6 months (T2) and 12 months (T3). Participants arrived at the ASU clinical research unit after an overnight fast. Height was measured to the nearest 0.1 cm using a portable stadiometer (SECA 213; SECA North America, Chino, CA) and weight was measured to the nearest 0.1 kg using an electronic scale (TBF300A; Tanita Corporation of America, Arlington Heights, IL). BMI and BMI percentiles were calculated for age and sex using the CDC growth charts. Glucose tolerance was determined via a 75‐g 2‐h OGTT, with glucose concentrations measured at fasting and 120 min.

### 2.3 Cost Calculations

We carried out a trial‐based cost effectiveness analysis of the Preventing Diabetes in Latino Youth study which accounted for resources spent and outcomes measured for the INT and UC arms over the 12‐month timeframe of the trial. Treatment cost from a health care payer perspective, cost from a family perspective and total societal cost (health care cost plus family cost) were included. Per‐person costs were calculated by dividing overall costs by the study sample. The reference year and currency for the analysis was 2019 US Dollars.

### 2.4 Health Care Perspective

The cost of delivering the INT and UC arms was determined using standard micro‐costing methods in which a detailed description of the costs required to replicate the treatment arms was accounted for and valued. The cost of the INT included personnel costs, staff training, staff travel, program venue, equipment and participant incentives. The cost of the UC included personnel costs, staff travel and program venue.

For the INT arm, the personnel costs for the YMCA trainers, community health educators and staff supervisor were estimated based on hourly wages and time spent. There was a total of 49 one‐hour sessions per participant in the 24‐week program (2 sessions per week and 1 orientation), with staff paid for 1.5 h (the additional 0.5 h was paid for preparation and clean‐up) per session. Staffs were also paid hourly wages for training sessions. Staff were paid for travel time to each session and reimbursed for mileage costs using the Internal Revenue Service (IRS) Standard Mileage Rates for 2019 (45.5 cents per mile). Mileage to each YMCA site was used to estimate fuel cost and travel time. Venue costs included a space usage fee for a group fitness room at the YMCA for the fitness sessions, as well as a classroom at the YMCA for the nutrition classes. Equipment costs included heart rate monitors, a unique weekly “food experience” in which healthy meals were served on‐site, and curriculum printing costs. Participant families were incentivised for attendance at intervention classes with YMCA memberships and earned incentives such as gift cards and sports or fitness equipment to use at home. All costs for the INT arm were accrued in the first 6 months, with no additional costs in the second 6 months.

For the UC arm, the personnel costs for the paediatric endocrinologist and the registered dietitian were estimated based on hourly wages and time spent. This included 1 h per participant with the physician and dietitian in the first 6 months of the trial, and an additional 1 h per participant with the physician and dietitian in the second 6 months of the trial. Physician and dietitian travel time costs were estimated for the UC in the same manner as the INT. Venue costs included a space usage fee for each 1‐h study visit.

### 2.5 Family Perspective

Parents completed questionnaires during baseline assessments in which they reported average income, the amount of work hours missed due to attending the INT sessions and round‐trip mileage to and from the INT or UC visits. Fuel cost was computed based on the 2019 IRS Standard Mileage Rates applied to the average round‐trip distance travelled to and from the INT sessions or the UC visits. Opportunity costs for lost wages were calculated for the INT arm by multiplying the average hourly participant wage rate by the average amount of work hours missed as reported by participants. For the UC arm, the physician/dietitian clinic visits all occurred during daytime working hours, and it was assumed that one parent lost 1 h of work time for each visit. Therefore, the opportunity cost for lost wages was calculated by multiplying the average participant hourly wage rate by 2 h (two 1‐h clinic visits).

### 2.6 Analytical Approach

To calculate incremental cost‐effectiveness ratios (ICERs), the difference in costs was divided by the changes in the health outcomes between the INT and UC groups. The health outcome used was the mean between‐group difference in change in 2‐h glucose level. As previously reported [20], 2‐h glucose efficacy end points were analysed using all randomly assigned participants per the intention‐to‐treat (ITT) principle. We used the full‐information maximum likelihood (FIML) estimation [22, 23] to allow for an ITT analysis that accounted for and adjusted for attrition. Attrition was 29% in the INT group and 11% in the UC group at 6 months, and 39% in the INT group and 32% in the UC group at 12 months. Using the FIML estimation ensured that all cases with valid data at baseline contributed to the estimates of intervention effects over time. The 2‐h glucose levels were treated so that reductions in this outcome were shown as positive values to indicate positive clinical changes and maintain consistency with conventions of interpretation of ICER values.

We performed a probabilistic sensitivity analysis (PSA) using 10 000 Monte Carlo simulations and constructed Cost‐Effectiveness Acceptability Curves (CEAC). In each simulation, program costs were sampled from a gamma distribution (mean = observed cost, SD = 10%) and 2‐h glucose reductions from a normal distribution (mean = observed effect, SD = observed SE). For each iteration, parameters are randomly drawn from their distributions to calculate incremental cost‐effectiveness ratios (ICERs) and then plotted against a range of willingness to pay (WTP) values ranging from $0 to $2000 per mg/dL 2‐h glucose reduction. The CEAC thus visually represents the uncertainty around the intervention's cost‐effectiveness, illustrating how changes in the WTP threshold affect the probability that the intervention is economically favourable compared with usual care. A cost‐effectiveness plane was also constructed by plotting incremental cost and effect values from the simulations.

Data analysis was conducted in SPSS statistical software version 27.0 (IBM Corp), MPlus version 8.7 (Muthén & Muthén) and Stata (StataCorp LLC).

## 3 Results

A total of 117 participants were enrolled and randomly assigned to a trial group, of whom 79 were assigned to INT and 38 to UC. Baseline anthropometric and biochemical characteristics are presented in Table 1.

**TABLE 1:** Baseline demographic, anthropometric and glycaemic characteristics.

Parameter	ALL (*n* = 117)	INT (*n* = 79)	UC (*n* = 38)
Age, year	13.5 ± 1.4	13.5 ± 1.3	13.6 ± 1.5
Female, *n* (%)	47 (40.1%)	33 (41.8%)	14 (36.8%)
Height, cm	164 ± 9	164 ± 9	164 ± 8
Weight, kg	91.3 ± 20.3	89.8 ± 18.3	94.5 ± 23.9
BMI, kg/m^2^	33.8 ± 5.4	33.3 ± 4.6	34.9 ± 6.7
BMI *z*‐score	2.28 ± 0.32	2.25 ± 0.30	2.33 ± 0.37
HbA1c, %	5.63 ± 0.28	5.63 ± 0.29	5.64 ± 0.27
Fasting Glucose,mg/dL	102 ± 8	101 ± 8	103 ± 7
2‐h Glucose, mg/dL	144 ± 30	144 ± 30	144 ± 29

*Note:* Data are presented as Mean ± SD for continuous variables.

Abbreviations: BMI, body mass index; HbA1c, haemoglobin A1c; INT, lifestyle intervention; UC, usual care.

Based on participant questionnaire results, the average participant wage was $9.52 per hour. The average amount of parental work hours missed due to participating in the INT was 3 h per participant. The average distance travelled from home to the INT session or the UC clinic visit was 7.0 miles.

As previously reported [20], at 6 months the INT led to a significant decrease in 2‐h glucose (144.5 ± 3.4 to 132.2 ± 3.4 mg/dL, Δ −12.3 mg/dL, *p* = 0.002) compared with baseline, while the UC led to a nonsignificant decrease in 2‐h glucose (144.1 ± 4.6 to 139.0 ± 4.6 mg/dL; Δ −5.1 mg/dL, *p* = 0.307). The difference in changes in 2‐h glucose between groups at 6 months was −7.2 mg/dL (95% CI, −19.7 to 5.3). At 12 months, within‐group reductions in 2‐h glucose among the INT (144.5 ± 3.4 to 129.1 ± 3.9 mg/dL, Δ −15.4 mg/dL, *p* = 0.002) and UC (144.1 ± 4.6 to 129.0 ± 4.7 mg/dL; Δ −15.1 mg/dL, *p* = 0.005) were significant from baseline, but did not differ between groups (0.3 mg/dL, 95% CI, −14.5 to 14.1).

A line‐itemised comparison of health care cost, family cost and total societal cost (health care cost plus family cost) for the intervention period (first 6 months, T1–T2), follow‐up period (second 6 months, T2–T3) and total trial (entire 12 months, T1–T3) is presented in Table 2. After 6 months, the estimated per‐participant cost from a health care perspective was $1838.25 (95% intervals, $1513.84–$2215.56) for the INT and $234.20 ($190.71–$278.61) for the UC. When family cost was included, the estimated per‐participant societal cost was $1935.21 ($1583.12–$2315.05) for the INT and $245.12 ($200.68–$296.86) for the UC.

**TABLE 2:** Health care cost, family cost and total societal cost after 6 and 12 months.

Parameter	Intervention (*n* = 79)						Usual care (*n* = 38)					
	Months 1–6	95% Interval	Months 7–12	95% Interval	Total	95% Interval	Months 1–6	95% Interval	Months 7–12	95% Interval	Total	95% Interval
Healthcare Cost (Payers)												
Physician Visit	—	—	—	—	—	—	$3458.00	—	$3458.00	—	$6916.00	—
Registered Dietician Visit	—	—	—	—	—	—	$1520.00	—	$1520.00	—	$3040.00	—
Intervention Personnel	$52189.50	—	—	—	$52189.50	—	—	—	—	—	—	—
Staff Travel	$3304.13	—	—	—	$3304.13	—	$691.60	—	$691.60	—	$1383.20	—
Venue	$53130.00	—	—	—	$53130.00	—	$3230.00	—	$3230.00	—	$6460.00	—
Staff Training	$1345.00	—	—	—	$1345.00	—	—	—	—	—	—	—
Equipment	$12975.00	—	—	—	$12975.00	—	—	—	—	—	—	—
Participant Incentives	22278.00	—	—	—	$22278.00	—	—	—	—	—	—	—
*Total Healthcare Cost*	$145221.63	—	$0	—	$145221.63	—	$8899.60	—	$8899.60	—	$17799.20	—
*Per Participant*	$1838.25	($1513.84–$2215.56)	$0	—	$1838.25	($1486.74–$2210.45)	$234.20	($190.71–$278.61)	$234.20	($190.92–$280.27)	$468.40	($379.19–$568.37)
Family Cost												
Fuel	$5403.92	—	—	—	$5403.92		$53.05	—	$53.05	—	$106.10	—
Treatment Time	$2256.24	—	—	—	$2256.24		$361.76	—	$361.76	—	$723.52	—
*Total Family Cost*	$7660.16	—	$0	—	$7660.16		$414.81	—	$414.81	—	$829.62	—
*Per Participant*	$96.96	($78.39–$116.13)	$0	—	$96.96	($79.63–$115.85)	$10.92	($8.93–$13.19)	$10.92	($8.87–$13.12)	$21.84	($17.80–$26.62)
Societal Cost												
*Total Cost*	$152881.79	—	$0	—	$152881.79		$9314.41	—	$9314.41	—	$18628.82	—
*Total Cost Per Participant*	$1935.21	($1583.12–$2315.05)	$0	—	$1935.21	($1562.30–$2327.47)	$245.12	($200.68–$296.86)	$245.12	($200.57–$299.98)	$490.24	($397.87–$592.01)

*Note:* “95% Interval” columns list the lower bound (2.5th percentile) and upper bound (97.5th percentile) of estimates. A dash (−) indicates that data are not applicable for each respective cell.

For the study follow‐up period (second 6 months), no additional costs were incurred for participants in the INT arm. Participants in the UC arm did incur additional costs, as the second visit with the paediatric endocrinologist and registered dietitian occurred during this time. At the completion of the entire 12 month study duration, the estimated per‐participant healthcare cost was $1838.25 ($1486.74–$2210.45) for the INT, while the estimated cost for the UC increased to $468.40 ($379.19–$568.37) per participant. When family cost was included, the estimated per‐participant societal cost for the INT group was $1935.21 ($1562.30–$2327.47) and $490.24 ($397.87–$592.01) for the UC group.

A summary of the ICER calculations at 6 months and 12 months is shown in Table 3. From a health care perspective, it cost $222.78 for an additional 1 mg/dL decrease in 2‐h glucose at 6 months in the INT group compared with the UC group. From a societal perspective, it cost $234.73 in the INT group for a 1 mg/dL decrease in 2‐h glucose relative to the UC group at 6 months. At 12 months, the ICER was $4566.17 per mg/dL for health care costs and $4816.57 per mg/dL for total societal costs for the INT compared with the UC.

**TABLE 3:** ICER calculations after 6 and 12 months.

Parameter	Incremental cost^a^	Change in 2‐h glucose^b^ (mg/dL)		Incremental effect^c^ (mg/dL)	ICER^d^
		INT	UC		
*After 6 months*					
Health Care Cost	$1604.05	−12.3 (5.0)	−5.1 (4.0)	−7.2 (−19.7, 5.3)	$222.78
Family Cost	$86.04	−12.3 (5.0)	−5.1 (4.0)	−7.2 (−19.7, 5.3)	$11.95
Total Societal Cost	$1690.09	−12.3 (5.0)	−5.1 (4.0)	−7.2 (−19.7, 5.3)	$234.73
*After 12 months*					
Health Care Cost	$1369.85	−15.4 (4.9)	−15.1 (5.4)	−0.3 (−14.5, 14.1)	$4566.17
Family Cost	$75.12	−15.4 (4.9)	−15.1 (5.4)	−0.3 (−14.5, 14.1)	$250.40
Total Societal Cost	$1444.97	−15.4 (4.9)	−15.1 (5.4)	−0.3 (−14.5, 14.1)	$4816.57

^a^ Incremental cost calculated using the difference in costs between INT and UC in Table 2.

^b^ Mean (SE).

^c^ Mean (95% CI).

^d^ ICER, Incremental Cost/Incremental Effect.

**FIGURE 1:**
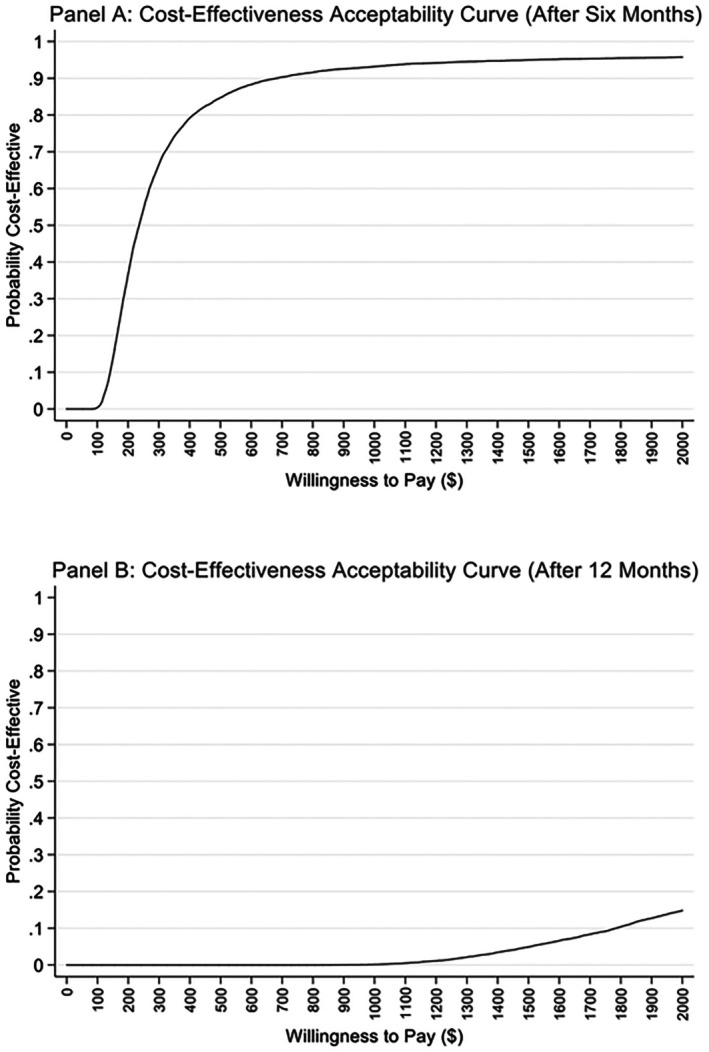
Cost effectiveness analysis acceptability curve at 6 and 12 months. The cost‐effectiveness acceptability curves (CEACs) in Figure 1 illustrate how the probability of intervention INT vs. UC being cost‐effective changes across different willingness‐to‐pay (WTP) thresholds at two‐time points: After 6 months (Panel A) and after 12 months (Panel B).

**FIGURE 2:**
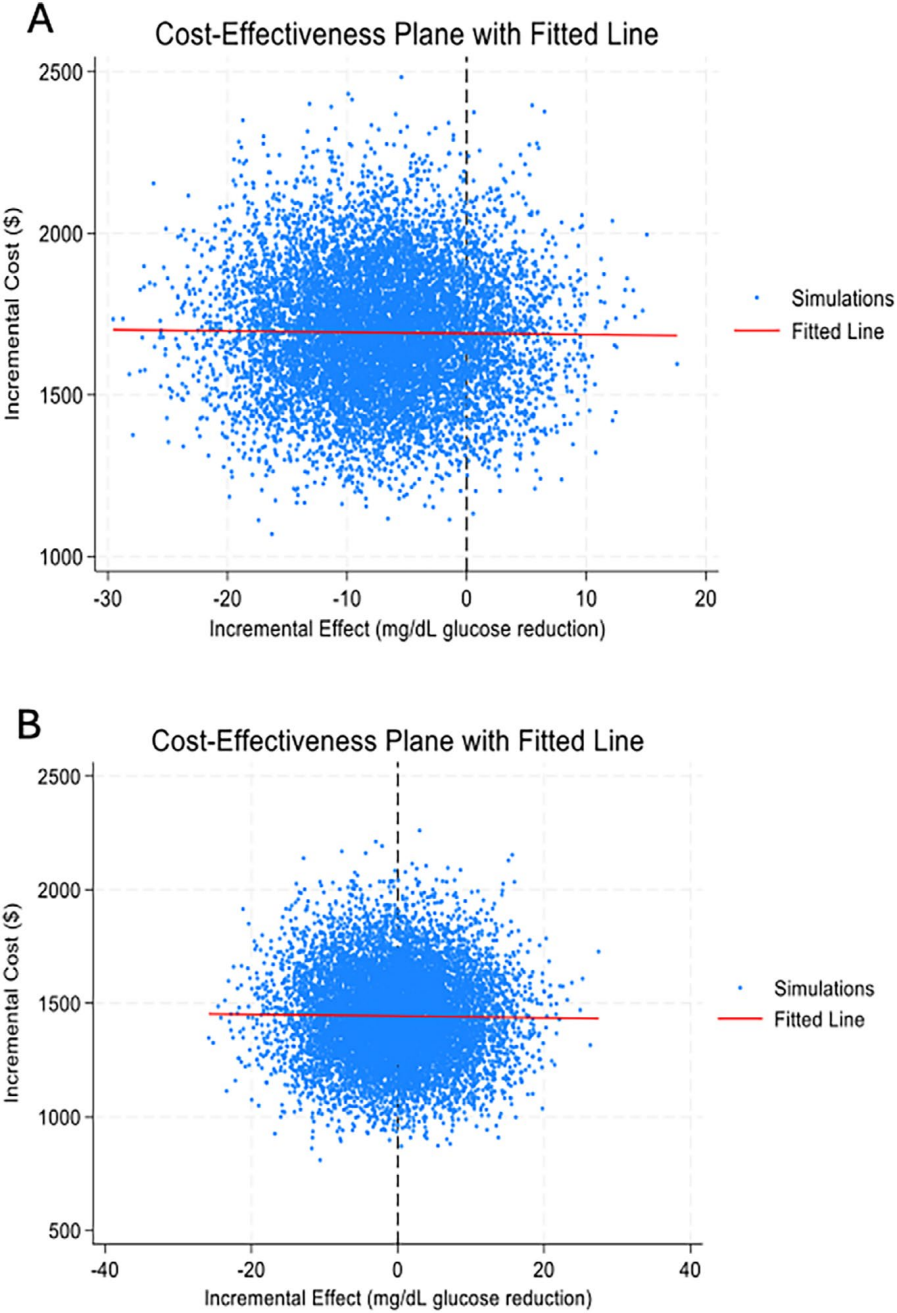
Cost effectiveness planes after 6 months (Panel A) and 12 months (Panel B). Figure 2 demonstrates no association between the incremental cost and incremental effectiveness.

Figure 1 demonstrates the CEAC for 2‐h glucose at 6 months (Panel A) and 12 months (Panel B). For Panel A, at low WTP thresholds (< $200), the probability of cost‐effectiveness is close to 0, meaning the intervention is unlikely to be cost‐effective if decision‐makers are unwilling to pay much. Therefore, after 6 months, the intervention demonstrates a high likelihood of cost‐effectiveness, particularly at WTP thresholds above $800, making it a favourable option for adoption. For Panel B, at low WTP thresholds (< $1000), the probability of cost‐effectiveness remains close to 0, indicating that the intervention is unlikely to be cost‐effective even with moderate WTP thresholds. Then the probability rises slightly after $1000 but remains below 20% at the maximum WTP of $2000. After 12 months, the intervention shows a much lower likelihood of cost‐effectiveness across all WTP thresholds, making it a less favourable option for implementation. For 6 months, we analysed the impact of a 10% increase and decrease in program cost ($1522 and $1860, respectively) compared with the actual cost of $1691.29, and adjusted the SE to 16.33 and 19.95, respectively, from the original SE of 18.14. For 12 months, similar adjustments were made: costs were varied to $1298 and $1586 (compared with the original cost of $1442.52), and SEs were modified to 11.96 and 14.62, compared with the original SE of 13.29. Despite these adjustments, the overall shape of the CEACs remained unchanged, suggesting that the results are robust to these variations in program costs and statistical uncertainty. As demonstrated in Figure 2, cost‐effectiveness planes demonstrated no relationship between the incremental effect and incremental costs after 6 months (Panel A) and 12 months (Panel B).

## 4 Discussion

Among an adolescent population at high risk for T2D, our study is the first to calculate the cost and incremental cost effectiveness of a diabetes prevention lifestyle intervention compared with usual care. The 6‐month intensive lifestyle intervention led to higher costs per 1 mg/dL reduction in 2‐h glucose compared with a usual care control from a total societal perspective. At 12 months compared with 6 months, the probability of cost effectiveness was decreased substantially, relative to usual care control.

In addition to demonstrating the DPP's clinical efficacy among high‐risk adults [12], further studies demonstrated its cost‐effectiveness and ability to reduce health care costs [9, 11]. This information contributed to the large‐scale implementation of the DPP as the National Diabetes Prevention Program by the Center for Disease Control and Prevention [24], a program that is now covered by Medicare [25] and Medicaid in some states. However, this robust and rigorous evidence base on effectiveness and cost‐effectiveness that shepherded the DPP to scale for high‐risk adults is lacking among high‐risk youth [26]; hence, it stands to reason that third‐party payer coverage of diabetes prevention lifestyle interventions does not exist for children and adolescents [27]. This important gap in the field hampers the ability of the public health sector and health systems to address the growing prevalence of T2D in the paediatric population—a growth projected to continue by the year 2060 with widening disparities among racial/ethnic populations [28]. To address this gap and guide implementation efforts, it is critical to understand the costs and cost‐effectiveness of lifestyle interventions among youth.

The importance of understanding both the economic consequences of health care interventions, as well as how to deliver health care efficiently, has become an increasing focus of health services researchers and policy makers [29]. Cost‐effectiveness analysis is an analytic tool in which the costs and health effects of an intervention and at least one alternative are measured and presented in a ratio of ICER [29]. It is ideal for cost‐effectiveness analyses to be reported by using a common health outcome that allows for comparisons across different types of treatments, such that criteria can be established regarding what constitutes a cost‐effective intervention [30]. The gold‐standard health outcome recommended for ICER calculations is cost per quality‐adjusted life year (QALY) gained [29]. The parent study included a measure of weight‐specific quality of life; however, this is not a preference‐based measure, no weights were available and therefore we were unable to assign a QALY. Instead, we chose improvement in 2‐h glucose as the health outcome measured in our ICER calculation, which was the parent study's primary outcome and an important marker of T2D risk. Given that 2‐h glucose in an ICER calculation lacks precedence in the health economic literature, there are no benchmarks available by which to make comparison to our results. Apart from QALYs, the next best metric would have been a primary health outcome such as the number of T2D cases prevented. However, this outcome is extremely difficult to measure in the paediatric population as paediatric T2D remains a rare diagnosis, and the sample size and follow‐up needed to differentiate T2D conversion rates would make such a study infeasible. We anticipate that other researchers may face similar challenges in measuring how paediatric diabetes prevention programs may affect QALY or other primary health outcomes, and thus we provide an example for future work to build on using an important clinical marker of T2D risk in the paediatric population.

The measurement of cost from a societal perspective is a challenging aspect of cost‐effectiveness analyses and is especially difficult in a paediatric population. The Second Panel on Cost‐Effectiveness in Health and Medicine recommended that all cost‐effectiveness studies “should report a reference case analysis based on a health care sector perspective and another reference case analysis based on a societal perspective” [29]. The societal perspective measures not just formal health care costs charged to third‐party payers or paid out‐of‐pocket by patients, but also includes costs related to patient and caregiver time, such as lost employment earnings and uncompensated household production. We attempted to account for lost parental employment earnings in our societal perspective analysis, however we acknowledge that the calculation of opportunity cost due to time spent in study activities requires significant assumptions for both study arms. For example, the intervention sessions were held in the evenings and therefore, time spent participating in the study could also have been spent in non‐work related activities such as leisure, household productivity, childcare, education and so on. Each of these alternative activities carries its own unique opportunity cost, albeit one that involves a high degree of interindividual variation. Therefore, additional opportunity costs to the participant and/or family as a result of participating in the study, including the loss of uncompensated household production, loss of school time and loss of entertainment and leisure time, were not able to be included.

An additional problem related to the measurement of family opportunity costs is the fact that while we attempted to account for parental opportunity costs associated with study activities, we were not able to capture the impact on health outcomes experienced by the parents (or other family members) related to their participation in either study arm, as these outcomes were not measured as part of the study. This likely led to an undervaluation of the cost‐effectiveness of the intervention relative to the usual care because at least one parent (and often other family members) actively participated in the lifestyle programme, which was designed to engage the entire family.

The high cost of the lifestyle programme compared with usual care bears some comments. The lifestyle intervention was much more time‐intensive than the usual care comparison, involving a total of 49 h versus 2 h, and this contributed to the intervention being far more costly than usual care. Although not focused exclusively on children with prediabetes, the US Preventive Services Task Force (USPSTF) found that intensive lifestyle interventions required at least 26 contact hours to improve weight status, and at least 52 contact hours to improve cardiovascular and metabolic health markers among youth with obesity [31]. The 26 and 52 contact hour targets require a substantial investment, both from the perspective of the health care system and the participant/family. While the USPSTF's Recommendation Statement provides an informative and much‐needed evidence‐based assessment of the efficacy of paediatric lifestyle interventions, it does not speak to the economic feasibility, practicality or sustainability of such high‐intensity interventions, all of which are key drivers to the successful implementation of a lifestyle intervention in a real‐world setting. Given the ever‐rising rates of obesity, prediabetes, type 2 diabetes and other obesity‐related co‐morbidities in the paediatric population, it is imperative that we not just evaluate the clinical efficacy of interventions to address paediatric obesity, but that we understand factors that will ensure effective implementation and sustainability as well.

This is the first study, to our knowledge, to undertake a cost‐effectiveness analysis of an intensive diabetes prevention lifestyle intervention in a paediatric population. A number of limitations are worth noting, starting with the lack of the use of QALYs as the health outcome metric in the ICER calculation, which precludes comparison of the cost‐effectiveness of our lifestyle intervention across other health domains. Additional limitations include the fact that our ICER calculations included costs incurred from study participants' parents/caregivers who participated in the intervention but did not account for improvements in health outcomes that the family members may have experienced. In addition, the small difference in the reductions in 2‐h glucose levels between intervention and control study arms was not statistically significant. To this end, changes in 2‐h glucose levels provide only a partial measure of the effect of the lifestyle intervention. For example, in the parent study, the intervention led to significant increases in weight‐specific quality of life compared with usual care [20]. Furthermore, due to the limited time duration of our study, we cannot account for health and healthcare cost benefits beyond 12 months. Therefore, because we analysed health outcomes from only a partial number of family members (one adolescent participant as opposed to the whole family who participated), performed the ICER calculation on one specific health outcome (2‐h glucose as opposed to other affected health measures such as quality of life), and were only able to measure health outcomes for 12 months, we suspect that our analysis does not capture the full impact of the intervention. Another limitation is that our sample size is small, which limits the precision of our estimated effect size.

In conclusion, a culturally grounded and community‐based lifestyle intervention among high‐risk Latino youth was more costly than usual care provided by a tertiary care paediatric obesity centre with appreciable, but non‐significantly different, reductions in 2‐h glucose over time. We are hopeful that our study provides an important step in moving the discussion forward regarding the economic value of diabetes prevention programmes in high‐risk youth.

## Author Contributions

M.L.O., A.P. and G.Q.S. were involved in the conception, design and conduct of the study. M.L.O., S.L.A. and A.B.S. conducted the analysis. M.L.O. wrote the first draft of the manuscript and all authors edited, reviewed and approved the final version of the manuscript. A.P. is the guarantor of this work and, as such, had full access to all the data in the study and takes responsibility for the integrity of the data and the accuracy of the data analysis.

## Conflicts of Interest

Dr. Olson reported receiving personal fees from Rhythm Pharmaceuticals outside the submitted work. Dr. Shaibi reported receiving personal fees from Phoenix Children's (PC) for research consultation, which has been disclosed to Arizona State University, outside the submitted work. PC participated in the research resulting in this publication. Dr. Peña received personal fees as an external reviewer at Novo Nordisk Foundation outside the submitted work.

## Data Availability

The datasets generated during and/or analysed in the current study are available from the corresponding author upon reasonable request.
